# Vitamin C content and profile of ascorbate metabolism gene expression in green leaves and bleached parts of the pseudostem of leek (Allium porrum L.) F1 hybrids

**DOI:** 10.18699/vjgb-25-23

**Published:** 2025-04

**Authors:** M.A. Filyushin, T.M. Seredin, A.V. Shchennikova, E.Z. Kochieva

**Affiliations:** Federal Research Centre “Fundamentals of Biotechnology” of the Russian Academy of Sciences, Moscow, Russia; Federal Research Centre “Fundamentals of Biotechnology” of the Russian Academy of Sciences, Moscow, Russia; Federal Research Centre “Fundamentals of Biotechnology” of the Russian Academy of Sciences, Moscow, Russia; Federal Research Centre “Fundamentals of Biotechnology” of the Russian Academy of Sciences, Moscow, Russia

**Keywords:** leek, Allium porrum L., vitamin C, ascorbate biosynthesis genes, ascorbate recycling genes, soluble sugars, gene expression, лук-порей, Allium porrum L., витамин С, гены биосинтеза аскорбата, гены рециклинга аскорбата, растворимые сахара, экспрессия генов

## Abstract

Leek (Allium porrum L.) is an economically important vegetable crop of the family Amaryllidaceae with a wide range of medicinal and nutritional properties, in part due to the accumulation of vitamin C (L-ascorbic acid, ascorbate). Ascorbate is an organic water-soluble compound, which performs many functions in plant cell metabolism, including as one of an effective antioxidant in plant cell responses to biotic and abiotic stress factors. Ascorbate metabolism includes biosynthesis (mainly the L-galactose pathway) and recycling (reduction of oxidized forms to ascorbate). The gene networks that determine ascorbate metabolism in leek plants are poorly understood. In this work, crosses of leek varieties/lines were carried out. Accessions of F1 hybrids were characterized for seed germination rate, ascorbate content and expression of ascorbate biosynthesis (PGI, PMI, PMM, VTC1b, GME1, GME2, VTC2, GPP, GalDH, GalLDH) and recycling (APX1, APX2, MDHAR1, MDHAR4, MDHAR5, DHAR2, GR) genes in seedlings, as well as green leaves and bleached stem parts of the adult plant. A search for correlations between the level of expression of ascorbate metabolism genes and the amount of vitamin C in leeks was also carried out. It was shown that the studied hybrids are characterized by high (89–100 %) seed germination, with the exception of the hybrid from the 74 × Alligator cross (55 %). An increased level of expression of the VTC2, MDHAR1, MDHAR4 and/ or MDHAR5 genes was detected in the seedlings and green leaves of nine F1 hybrids, which allowed us to consider these samples promising in terms of possible stress resistance. Four hybrids that were characterized by the lowest (33 × 30, 74 × Alligator) and highest (81 × 95, 36 × 38) ascorbate content in seedlings were selected for a further detailed analysis of adult plants for the content of soluble sugars and ascorbate, gene expression and morphological characteristics (length, thickness and weight of the false stem). It was confirmed that green leaves of the 36 × 38 and 81 × 95 hybrids contain significantly more ascorbate than the 33 × 30 and 74 × Alligator hybrids. In all four hybrids, the ascorbate content was significantly lower in the bleached stems than in the green leaves. Accessions 36 × 38 and 81 × 95 were also characterized by the highest amount of soluble sugars in the bleached part of the false stem used for food. In addition, the false stem formed by the 81 × 95 hybrid was larger and heavier than the stems of the other three hybrids. A direct dependence of ascorbate content on the transcript level of ascorbate recycling genes (APX2, MDHAR1, MDHAR4) in green leaves was revealed, which can be used in the breeding of stress-resistant leek hybrids with a high content of vitamin C.

## Abstract (ru)

Лук-порей (Allium porrum L.) – экономически важная овощная культура семейства Amaryllidaceae с широким спектром лечебных и питательных качеств, в том числе благодаря накоплению витамина С (L-аскорбиновая кислота, аскорбат). Аскорбат – органическое водорастворимое соединение, которое выполняет множество функций в метаболизме клеток растения, в том числе участвует в качестве эффективного антиоксиданта в ответе растительных клеток на биотические и абиотические стрессовые факторы. Метаболизм аскорбата включает биосинтез (преимущественно L-галактозный путь) и рециклинг (восстановление окисленных форм до аскорбата). Генные сети, определяющие метаболизм аскорбата в растениях лука-порея, малоизученны. В настоящей работе гибриды F1, полученные от 13 скрещиваний образцов лука-порея отечественной и зарубежной селекции, охарактеризованы по всхожести семян, содержанию аскорбата и экспрессии генов биосинтеза (PGI, PMI, PMM, VTC1b, GME1, GME2, VTC2, GPP, GalDH и GalLDH) и рециклинга (APX1, APX2, MDHAR1, MDHAR4, MDHAR5, DHAR2, GR) аскорбата в проростках, зеленых листьях и отбеленной части стебля взрослых растений. Также проведен поиск корреляций между уровнем экспрессии генов метаболизма аскорбата и количеством витамина С у лука-порея. Показано, что изучаемым гибридам присуща высокая (89–100 %) всхожесть семян, за исключением гибрида от скрещивания 74 × Аллигатор (55 %). В проростках и зеленых листьях образцов девяти гибридов F1 выявлен повышенный уровень экспрессии генов VTC2, MDHAR1, MDHAR4 и/или MDHAR5, что позволило считать эти образцы перспективными с точки зрения возможной стрессоустойчивости. Четыре гибрида, которые характеризовались наименьшим (33 × 30, 74 × Аллигатор) и наибольшим (81 × 95, 36 × 38) содержанием аскорбата в проростках, были выбраны для дальнейшего детального анализа взрослых растений на содержание растворимых сахаров и аскорбата, уровень экспрессии генов метаболизма аскорбата и морфологические характеристики (длина, толщина и масса ложного стебля). Выявлено, что зеленые листья гибридов 36 × 38 и 81 × 95 содержат существенно больше аскорбата, чем 33 × 30 и 74 × Аллигатор. В отбеленных стеблях всех четырех гибридов содержание аскорбата было значительно ниже, чем в зеленых листьях. Образцы 36 × 38 и 81 × 95 отличались также наибольшим количеством растворимых сахаров в отбеленной части ложного стебля, употребляемой в пищу. Гибрид 81 × 95 формировал ложный стебель, превышающий по размеру и массе стебель осталь- ных трех гибридов. В зеленых листьях содержание аскорбата положительно коррелировало с уровнем экспрессии генов рециклинга аскорбата (APX2, MDHAR1, MDHAR4), что может быть использовано в селекции стрессоустойчивых гибридов лука-порея с повышенным содержанием витамина С.

## Introduction

Onion species, including leek (Allium porrum L.), have a wide range of nutritional and medicinal properties. Leek, which is also known as A. ampeloprasum var. porrum (L.) Gay, is considered an economically important vegeTable crop valued not only for its nutritional qualities but also for its antibacterial, anticancer, cardioprotective, and antioxidant properties (Celebi-Toprak, Alan, 2021).

Leek breeding is aimed at increasing the length, thickness, density and weight of the edible white (blanched) stem, improving its taste and dietary qualities, as well as increasing seed germination and resistance to stress factors and bolting (Swamy, Gowda, 2006; Celebi-Toprak, Alan, 2021). Soluble sugars (5.0–11.2 g/100 g raw weight) and vitamin C (L-ascorbic acid, ascorbate, AA) (0.9–3.6 mg/g dry weight) give leeks a delicate and sweet taste. During storage, the amount of AA in the blanched part of the false stem increases by more than 1.5 times (Lundegårdh et al., 2008; Grzelak-Błaszczyk et al., 2011; Bernaert et al., 2012; Bernaert, 2013). Both types of metabolites play significant roles in the plant’s defense responses to stress factors (Yamada, Osakabe, 2018; Broad et al., 2020; Qi et al., 2020), and vitamin C is also important for human health (Hemilä, 2017).

The presence of ascorbate is also positively associated with the post-harvest shelf life of the blanched stem, since, unlike onions, the cut stem of leeks is not in a state of physiological dormancy and quickly deteriorates (Bernaert, 2013). Furthermore, ascorbate and soluble sugar-dependent signaling pathways largely determine plant ontogeny (Considine, Foyer, 2014; Yoon et al., 2021) and may therefore positively influence leek pseudostem size.

A comparison of oil extracts of leeks and another equally popular onion crop, garlic (A. sativum L.), showed the superiority of leeks in antioxidant activity, largely due to higher accumulations of vitamin C (Lemma et al., 2022). According to several studies, the amount of vitamin C in green leaves and the edible blanched part of the stem (false stem) of leeks can vary within 2.8–8.5 and 0.9–3.6 mg/g dry weight, respectively (Lundegårdh et al., 2008; Bernaert et al., 2012).

Vitamin C is an organic water-soluble compound that is not synthesized by humans, but is a necessary part of human diet and comes from plant foods, where the amount of ascorbate depends on the species/variety, tissue/organ, and plant growing/ storage conditions (Bulley, Laing, 2016). In addition to its benefits to humans, ascorbate is involved in many aspects of plant development, including the regulation of cellular metabolism, and is also an effective antioxidant, since it is present in the cell in sufficient quantities and carries out fine regulation of the presence of various free radicals, reacting with them (Arrigoni, De Tullio, 2002).

The importance of ascorbate for the plant is emphasized by the fact that its synthesis occurs through several unique pathways, the dominant of which is the Smirnov–Wheeler L- galactose pathway, which undergoes eight stages of conversion of the initial substrate (D-fructose-6-P) into L-ascorbic acid (Bulley, Laing, 2016). The biosynthetic pathway includes reactions catalyzed sequentially by glucose-6-phosphate isomerase (PGI), mannose-6-phosphate isomerase (PMI), phosphomannomutase (PMM), GDP-mannose pyrophosphorylase (VTC1), GDP-mannose 3′,5′-epimerase (GME), GDP-L-galactose phosphorylase (VTC2, VTC5), L-galactose- 1-phosphate phosphatase (GPP), L-galactose dehydrogenase (GalDH), and L-galactono-1,4-lactone dehydrogenase (GalLDH) (Bulley, Laing, 2016).

Ascorbate recycling occurs as follows. When interacting with active forms of oxygen, as well as under the action of ascorbate peroxidases (APX) and ascorbate oxidases (AO), ascorbate is oxidized and converted into monodehydroascorbic acid (MDHA), which can be broken down into dehydroascorbic acid (DHA) and ascorbate (Bulley, Laing, 2016). Both oxidized forms (MDHA and DHA) can be reduced to ascorbate by monodehydroascorbate reductase (MDHAR) and dehydroascorbate reductase (DHAR), respectively (Bulley, Laing, 2016). Thus, the concentration of ascorbate in plant tissue is determined by the balance between the synthesis of vitamin C, its recycling and the catabolism of oxidized forms.

Gene networks of ascorbate metabolism are studied in various plant species, including cultivated species. For example, the expression level of the VTC2 gene has been shown to be positively associated with the amount of ascorbate in plant tissue and with plant resistance to abiotic stress factors; this fact is used in breeding aimed at increasing the vitamin C content (Ali et al., 2019; Broad et al., 2020). In the model species Arabidopsis thaliana L., a paralog of VTC2, the VTC5 gene, was found, but the rate of the L-galactose pathway is determined predominantly by the activity of VTC2 (Dowdle et al., 2007).

Ascorbate recycling genes are studied more in terms of their role in determining plant stress tolerance. Exposure to various stress factors (both abiotic and biotic) leads to changes in the expression level of MDHAR genes and the activity of the enzymes they encode (Leterrier et al., 2005; Dowdle et al., 2007; Gill, Tuteja, 2010; Feng et al., 2014; Lanubile et al., 2015; Zhang et al., 2015; García et al., 2020). Overexpression of MDHAR genes has a positive effect on salt stress tolerance (Sultana et al., 2012; Qi et al., 2020). However, in ripe tomato fruits, this significantly reduces the ascorbate content (Haroldsen et al., 2011).

Gene networks determining ascorbate metabolism in leek plants (A. porrum) are poorly studied. The polymorphism and expression profile of the VTC2 gene have been characterized, including in response to cold stress, and a correlation between the VTC2 expression level and the ascorbate content in green leaves (positive) and the white part (negative) has been shown (Anisimova et al., 2021a, b). Three MDHAR genes have been identified and characterized; the level of transcripts of one of them, MDHAR4, positively correlates with the content of AA in the white part and green leaves of the plant (Filyushin et al., 2021). No other publications on the characterization of AA metabolism genes in leeks have been found.

The aim of this work was to obtain F1 leek hybrids from 13 crosses of leek accessions of domestic and foreign selection and compare them by vitamin C content and the expression level of genes of AA biosynthesis (PGI, PMI, PMM, VTC1b, GME1, GME2, VTC2, GPP, GalDH, GalLDH) and recycling (APX1, APX2, MDHAR1, MDHAR4, MDHAR5, DHAR2, GR) in sprouts, green leaves and the blanched part of the stem of adult plants. A possible correlation between the expression level of the analyzed genes and the amount of vitamin C in leek tissues was assessed.

## Materials and methods

In the study, we used seeds of F1 hybrids obtained from 13 crosses (2022) of leek varieties/lines of domestic and foreign selection (Table 1). F1 seeds were sown (50 pcs. from each cross) in the soil (experimental climate control facility, FRC Biotechnology RAS; day/night – 16 h/8 h, 23 °C/21 °C), germination was assessed, and the resulting plants were used in further analysis

**Table 1.**
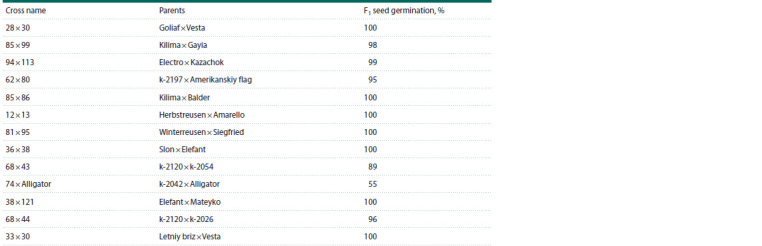
List of crosses of leek accessions

The aboveground part of the seedlings (30 days after germination) of the F1 hybrids as well as green leaves and blanched false stems of adult plants at the commercial stage of development were used in further analysis

The commercial stage of development is understood as plants before the flowering phase, the growth of which is complete, and the length and diameter of the stem have reached final dimensions. Samples of plant tissue were ground in liquid nitrogen and used to determine the content (mg/100 g raw weight) of vitamin C, sucrose, glucose and fructose using the Enzytec L-Ascorbic Acid, Enzytec™ Liquid Sucrose/D- Glucose and Enzytec™ Liquid D-Glucose/D-Fructose (R-Biopharm AG, Germany) kits, following the protocols provided by the manufacturer. Each sample type (sprout, green leaf or false stem) was ground whole and stored at –80 °C, taking the required portion for the analysis (determination of ascorbate concentration or gene expression).

Analysis of expression of the L-galactose biosynthetic pathway genes and the ascorbate recycling pathway genes was performed using quantitative real-time PCR (qRT-PCR). Total RNA was isolated from 0.2–0.5 g of ground tissue using the RNeasy Plant Mini Kit (QIAGEN, Germany). DNA impurities were removed using the RNase-free DNase set (QIAGEN, Germany), and cDNA was synthesized in the GoScript Reverse Transcription System (Promega, USA). The concentration of cDNA was determined using the Qubit® Fluorometer (Thermo Fisher Scientific, USA) and Qubit RNA HS Assay Kit (Invitrogen, USA), and 3 ng of the preparation was used in the qRT-PCR reaction. Gene-specific primers for qRT-PCR were designed based on the genomic/transcriptomic data of A. porrum (PRJNA310797) and A. sativum (PRJNA606385, PRJNA607255) available at NCBI (Table 2).

**Table 2.**
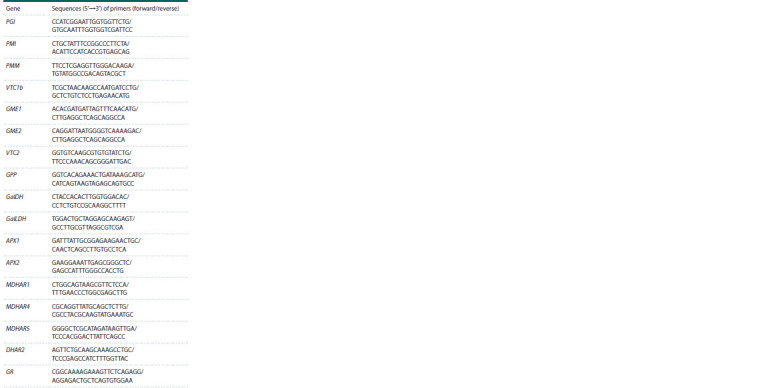
Sequences of primers used in this work for qRT-PCR analysis

The reaction used the “2.5× Reaction mixture for qRT-PCR in the presence of SYBR Green I and ROX” kit (Synthol, Russia). qRT-PCR was performed in a CFX96 Real-Time PCR Detection System (Bio-Rad Laboratories, USA) using the program: 5 min at 95 °C, 40 cycles (15 s at 95 °C; 40 s at 60 °C). To normalize gene expression, two references were used: the glyceraldehyde-3-phosphate dehydrogenase (GAPDH) and Ubiquitin (UBQ) genes (Anisimova et al., 2021a). The analysis was performed in two biological and three technical replicates. The results were processed and correlation analysis was performed in GraphPad Prism v. 9.5.1 (GraphPad Software Inc., USA; https://www.graphpad.com/ scientific-software/prism/).

Morphological characteristics (length, thickness and weight of the blanched part (false stem)) of leek plants were assessed in September 2023, using 10 plants of each analyzed hybrid. The plants were grown in an onion collection nursery (greenhouse coordinates 55.655182, 37.206576; Federal Scientific Vegetable Center, Moscow Region, Ph.D. in Agricultural Sciences T.M. Seredin).

## Results

In this work, 13 F1 hybrids obtained from crossing domestic and foreign leek varieties/lines were characterized (Table 1).

**Fig. 1.**
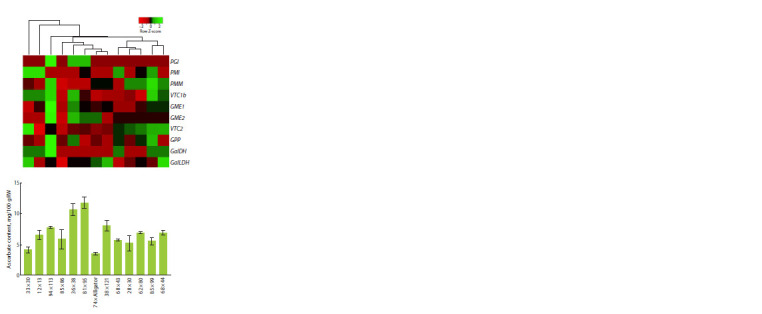
Heatmap of L-galactose ascorbate biosynthetic pathway gene expression (PGI, PMI, PMM, VTC1b, GME1, GME2, VTC2, GPP, GalDH, GalLDH) and vitamin C content (mg/100 g raw weight) in seedlings of F1 hybrids from 13 crosses of leek varieties/lines.

**Fig. 2.**
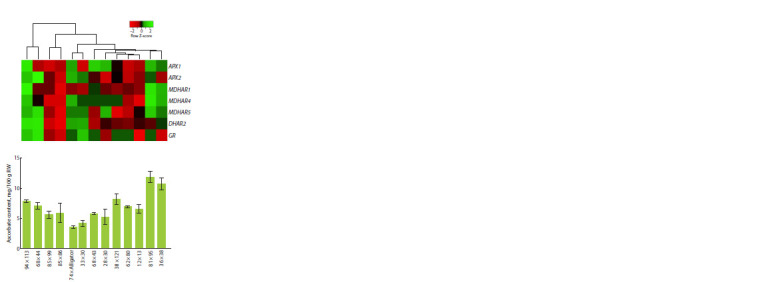
Heatmap of ascorbate recycling gene expression (APX1, APX2, MDHAR1, MDHAR4, MDHAR5, DHAR2, GR) and vitamin C content (mg/100 g raw weight) in seedlings of F1 hybrids from 13 crosses of leek varieties/lines.

F1 sprouts were characterized by the ascorbate content and the expression level of genes of the L-galactose pathway of ascorbate biosynthesis and its recycling pathway (Fig. 1, 2).

The highest amount of vitamin C was found in sprouts from crossings 36 × 38 and 81 × 95, the lowest – 33 × 30 and 74 × Alligator, while the remaining accessions showed intermediate values (5–7.5 mg/100 g raw weight) (Fig. 1).

On the heatmap of ascorbate synthesis gene expression, accessions 36 × 38 and 81 × 95 occupied adjacent positions in a cluster that also included hybrids with medium (85 × 86, 38 × 121) and the lowest (74 × Alligator) ascorbate content. Five accessions (68 × 43, 28 × 30, 62 × 80, 85 × 99, 68 × 44), characterized by similar average ascorbate content, formed a separate cluster (Fig. 1).

On the heatmap of ascorbate recycling gene expression, pairs of hybrids with the highest (36 × 38, 81 × 95) and lowest (33 × 30, 74 × Alligator) vitamin C content formed two separate clusters. We also note two clusters (94 × 113/68 × 44 and 85 × 99/85 × 86), the accessions in which were highly similar in gene expression and ascorbate content (Fig. 2).

For further analysis, we selected crosses contrasting in the amount of vitamin C in seedlings: 36 × 38 and 81 × 95 (the highest); 33 × 30 and 74 × Alligator (the lowest). In September 2023, 10 adult F1 plants from these crosses were characterized by ascorbate content and the expression level of genes of the L-galactose pathway of ascorbate biosynthesis and ascorbate recycling genes in green leaves and the blanched false stem.

**Fig. 3.**
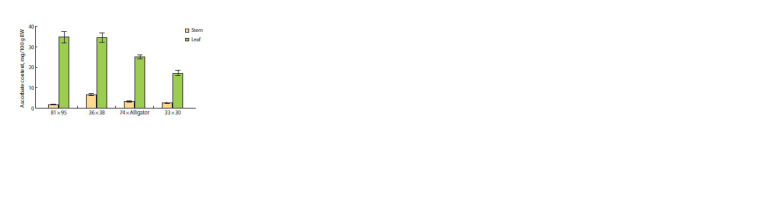
Ascorbate content in the blanched false stem and green leaves of adult plants of F1 leek hybrids from the crosses 81 × 95, 36 × 38, 74 × Alligator and 33 × 30.

As a result, it was found that the green leaves of the 36 × 38 and 81 × 95 plants contained a similar (~35 mg/100 g raw weight) amount of ascorbate, which was expected to be significantly higher than that of the 33 × 30 and 74 × Alligator plants (~25 and ~17 mg/100 g raw weight, respectively). In the blanched false stems of all four types of plants, the ascorbate content was significantly lower than in the green leaves and did not exceed 6.5 mg/100 g raw weight. At the same time, the 33 × 30 and 74 × Alligator plants showed similar average values, and the remaining two hybrids were characterized by the lowest (81 × 95) and highest (36 × 38) amount of vitamin C (Fig. 3)

**Fig. 4.**
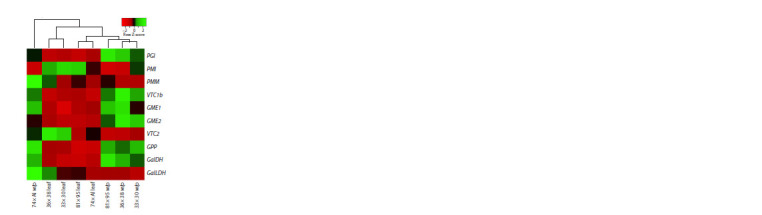
Heatmap of the expression of genes of the L-galactose pathway of ascorbate biosynthesis in the white part (w/p) and leaf tissue of F1 hybrid leek plants from the crosses 81 × 95, 36 × 38, 74 × Alligator (Al) and 33 × 30.

Gene expression analysis showed that in all four hybrids, the expression level of ascorbate biosynthesis genes in the blanched part of the false stem was predominantly higher than in the green leaves, with the exception of the PMI, PMM, VTC2 and GalLDH genes (Fig. 4). No dependence of hybrids clustering on the expression heatmap on the ascorbate content (Fig. 3) was observed (Fig. 4).

**Fig. 5.**
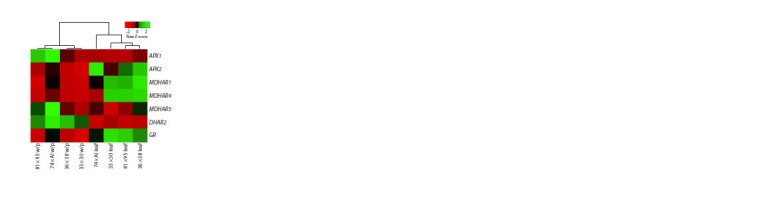
Heatmap of the expression of genes of the ascorbate recycling pathway in the white part (w/p) and green leaves of F1 hybrid leek plants from the crosses 81 × 95, 36 × 38, 74 × Alligator (Al) and 33 × 30.

**Fig. 6.**
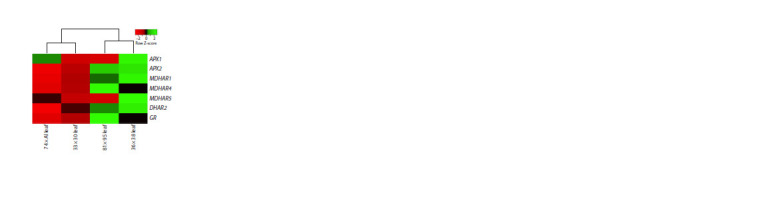
Heatmap of the expression of genes of the ascorbate recycling pathway in the green leaves of F1 hybrid leek plants from the crosses 74 × Alligator (Al), 33 × 30, 81 × 95 and 36 × 38.

The highest transcript level of ascorbate recycling genes was found in green leaves of the analyzed plants, except for the APX1, MDHAR5, and DHAR2 genes with higher expression in the false stem (Fig. 5). At the same time, the heatmap clearly demonstrated the formation of two clusters, combining false stems and green leaves, respectively (Fig. 5). The “leaf” cluster included hybrids with an increased content of vitamin C in the leaves (36 × 38 and 81 × 95), and when clustering only by the level of gene expression in the leaves, the hybrids were strictly divided into groups with high (36 × 38 and 81 × 95) and low (74 × Alligator, 33 × 30) vitamin C content (Fig. 6). Moreover, the leaves of the 36 × 38 and 81 × 95 plants (in comparison with the 74 × Alligator and 33 × 30 hybrids) were distinguished by a significantly higher expression of ascorbate recycling genes, with rare exceptions (APX1 and MDHAR5 in the 81 × 95 plants) (Fig. 6).

In the case of the “false stem” cluster, no dependence of hybrids grouping on the amount of ascorbate was observed. For example, the 74 × Alligator and 33 × 30 plants were assigned to separate groups (Fig. 5), despite similar vitamin C content (Fig. 3).

**Fig. 7.**
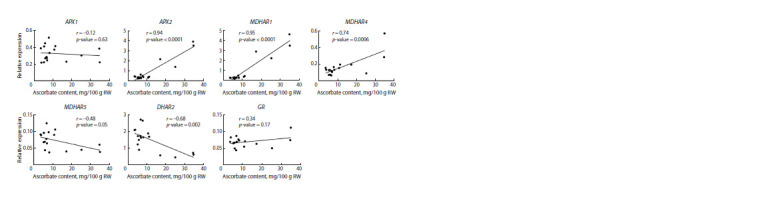
Linear regression of vitamin C content and expression levels of ascorbate recycling pathway genes in green leaves of F1 leek hybrids. r is the Pearson correlation coefficient (correlation at p < 0.01).

Next, using seedling and adult plant data, we searched for possible correlations between ascorbate content and gene expression levels, and revealed a high correlation for the ascorbate recycling genes APX2, MDHAR1, and MDHAR4 (r = 0.94, 0.95, and 0.74, respectively) in leaves (Fig. 7).

**Fig. 8.**
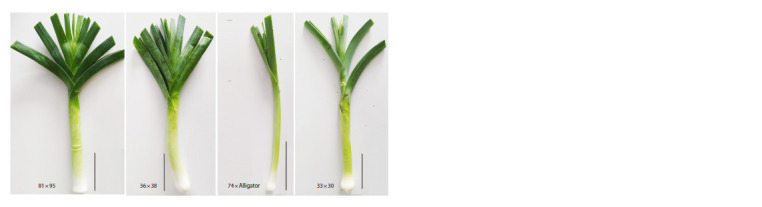
The commercial part of F1 hybrids from the crosses 81 × 95, 36 × 38, 74 × Alligator and 33 × 30. Scale = 10 cm.

The four analyzed hybrid lines were characterized by their morphological features. It was shown that the 81 × 95 hybrid forms a strong false stem 25–30 cm long, 3.5–5 cm thick, weighing 250–350 g with dense leaf arrangement. The 36 × 38 hybrid has a strong false stem 20–25 cm long, 3–4.5 cm thick, weighing 200–300 g with dense leaf arrangement (Fig. 8). Both hybrids are characterized by 100 % seed germination (Table 1).

The false stem of the 74 × Alligator hybrid is long (20– 25 cm) and thin (1.5–2.5 cm), weighing 100–150 g; the bulb is pronounced; the plants are significantly slower in growth than the other hybrids studied. The 33 × 30 hybrid is characterized by a powerful, loose false stem 20–25 cm long, 2.5–3.5 cm thick, and weighing 150–250 g; the bulb is pronounced (Fig. 8). The hybrids are characterized by 55 % (74 × Alligator) and 100 % (33 × 0) seed germination (Table 1).

**Fig. 9.**
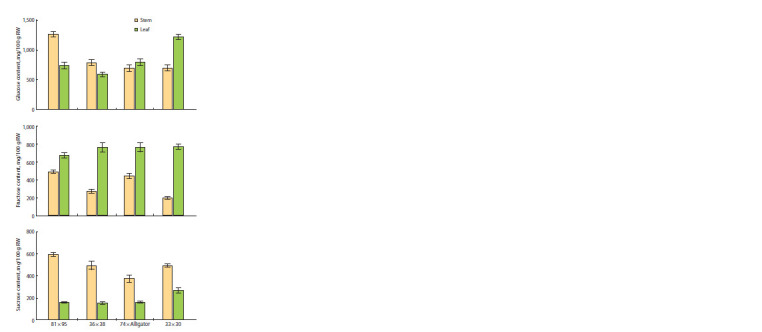
Content of glucose, fructose and sucrose in the false stem and leaves of adult plants of F1 leek hybrids from the crosses 81 × 95, 36 × 38, 74 × Alligator and 33 × 30.

The analyzed hybrids were also additionally characterized by the content of soluble sugars in green leaves and the blanched part of the false stem. It was shown that, compared to the leaves, the false stem is enriched with sucrose and contains ~1.5–3 times less fructose (Fig. 9). The amount of glucose turned out to be more variable: similar between leaves and stem (36 × 38, 74 × Alligator); the highest in the stem (81 × 95); the highest in the leaves (33 × 30) (Fig. 9). Considering the blanched part of the stem used for food, it can be seen that the highest amounts of all three sugars are contained in the F1 hybrid from the cross 81 × 95; the least amount of fructose – in 33 × 30, and that of sucrose – in 74 × Alligator; the glucose content is similar in three hybrids (36 × 38, 33 × 30, 74 × Alligator) (Fig. 9).

## Discussion

In this study, 13 F1 leek hybrids with seed germination above 50 % (Table 1) were characterized for ascorbate content and expression of genes for ascorbate biosynthesis and recycling in seedlings (Fig. 1, 2). Variations in vitamin C content within 30 % between hybrids (Fig. 1) were consistent with previously demonstrated differences in ascorbate concentration in green leaves and false stems of leek (Lundegårdh et al., 2008; Bernaert et al., 2012).

The resulting selected accessions of two F1 hybrids (81 × 95, 36 × 38), promising due to the highest vitamin C content, were further characterized at the commercial stage of development in comparison with two F1 hybrids (74 × Alligator, 33 × 30) with the lowest vitamin C content. Characterization included analysis of ascorbate/soluble sugar content and expression of ascorbate metabolism genes in green leaves and the blanched part of the pseudostem, as well as morphological description (Fig. 3–9).

It was shown that the results obtained from the analysis of ascorbate content in sprouts could be used to evaluate this trait only in the case of green leaves of adult plants, while the blanched part of the false stem shows variable data (Fig. 3). One of the promising hybrids (36 × 38) showed the highest content of vitamin C in the false stem, while the second hybrid (81 × 95), on the contrary, contained the lowest amount of ascorbate among the four accessions (Fig. 3).

Comparison of the ascorbate content data and clustering of the studied hybrids by the expression profile of the vitamin C synthesis genes (Fig. 4) showed that the expression level of any gene in the pathway could not be used to draw conclusions about the amount of ascorbate in the leaves or stems of leeks. In particular, the expression level of the VTC2 gene (Fig. 1, 4), for which a relationship with ascorbate content was previously proposed (Ali et al., 2019; Broad et al., 2020), including in leeks (Anisimova et al., 2021a), is not suitable for prediction.

Nevertheless, the level of VTC2 gene transcripts that we determined in hybrids can help to predict the degree of stress resistance of the accessions, since the level of VTC2 expression is positively associated with plant resistance to abiotic stress factors (Ali et al., 2019; Broad et al., 2020). Based on our results (expression in seedlings or green leaves (Fig. 1, 4)), six promising hybrids can be identified, among which are two of the four selected for analysis (the studied 33 × 30 and 36 × 38, as well as 28 × 30, 62 × 80, 85 × 99, 68 × 44).

Comparison of biochemical data and clustering of hybrids by the expression of ascorbate recycling genes (Fig. 5, 6) allowed us to suggest a dependence of the level of ascorbate accumulation in leek leaves or stems on the expression profile of the pathway genes. In addition, since increased activity of MDHAR genes and the enzymes they encode is associated with plant stress resistance (e. g., Zhang et al., 2015; Qi et al., 2020), based on our data on three MDHAR genes (Fig. 2, 5, 6), we can identify seven promising hybrids, among which are all four accessions selected for analysis (studied 81 × 95, 36 × 38, 33 × 30, 74 × Alligator, as well as 94 × 113, 68 × 44, 28 × 30).

Our assessment of possible statistically significant correlations between ascorbate content and the expression level of ascorbate metabolism genes revealed correlations only in green plant tissue (seedlings, green leaves) and only for three ascorbate recycling genes: APX2, MDHAR1 and MDHAR4 (Fig. 7). This confirms our previously identified positive relationship between the MDHAR4 transcript level and the vitamin C content in leek plants (Filyushin et al., 2021).

The characteristics of the content of soluble sugars in green leaves and the blanched part of the false stem of the studied hybrids showed the absence of any dependence on the concentration of ascorbate (Fig. 9). Nevertheless, taking into account the obtained data, it can be assumed that the blanched part of the false stem in the F1 81 × 95 hybrid, which is also characterized by the highest amount of ascorbate, has a greater nutritional value.

The morphological characteristics of the analyzed F1 hybrids showed that the largest amounts of sugars (Fig. 9) correspond to the largest size and weight of the false stem in the hybrid from the 81 × 95 cross. The other three hybrids, taking into account the average data for all three types of sugars in the blanched part (a total of 1,500–1,600 mg/100 g raw weight (Fig. 9)), correspond to a similar length of the false stem (20–25 cm vs. 25–30 cm in 81 × 95). However, the variations in other stem parameters (thickness, weight) in these three hybrids do not agree in any way with either the amount of sugars or the content of each individual type of sugar.

## Conclusion

Thus, the performed characterization of F1 leek hybrids from 13 crosses made it possible to select nine accessions promising in terms of stress resistance (81 × 95, 36 × 38, 33 × 30, 74 × Alligator, 94 × 113, 28 × 30, 62 × 80, 85 × 99, 68 × 44). Of these, eight hybrids showed 95–100 % seed germination (except for 74 × Alligator, 55 %). Two hybrids (81 × 95, 36 × 38) were characterized by the highest ascorbate content in the green tissue (sprouts, green leaves) of the plant and one (36 × 38) – in the blanched part of the false stem, used as food. Hybrid 81 × 95 also accumulated the highest amount of soluble sugars in the blanched part. The found direct dependence of ascorbate content on the activity of ascorbate recycling genes (APX2, MDHAR1, MDHAR4) in green leaves can be used in the breeding of stress-resistant hybrids with increased vitamin C content. Further studies are needed on the possible relationship between the expression level of the APX2, MDHAR1, MDHAR4 and VTC2 genes and plant resistance to various stress factors, the results of which can be used in the breeding of stress-resistant leek hybrids.

## Conflict of interest

The authors declare no conflict of interest.
